# Interorganelle Communication between Mitochondria and the Endolysosomal System

**DOI:** 10.3389/fcell.2017.00095

**Published:** 2017-11-07

**Authors:** Gonzalo Soto-Heredero, Francesc Baixauli, María Mittelbrunn

**Affiliations:** ^1^Centro de Biología Molecular Severo Ochoa (CSIC-UAM), Madrid, Spain; ^2^Max Planck Institute of Immunobiology and Epigenetics, Freiburg, Germany; ^3^Instituto de Investigación Hospital 12 de Octubre, Madrid, Spain

**Keywords:** lysosome, exosomes, autophagy, quality control, proteostasis, aging

## Abstract

The function of mitochondria and lysosomes has classically been studied separately. However, evidence has now emerged of intense crosstalk between these two organelles, such that the activity or stress status of one organelle may affect the other. Direct physical contacts between mitochondria and the endolysosomal compartment have been reported as a rapid means of interorganelle communication, mediating lipid or other metabolite exchange. Moreover, mitochondrial derived vesicles can traffic obsolete mitochondrial proteins into the endolysosomal system for their degradation or secretion to the extracellular milieu as exosomes, representing an additional mitochondrial quality control mechanism that connects mitochondria and lysosomes independently of autophagosome formation. Here, we present what is currently known about the functional and physical communication between mitochondria and lysosomes or lysosome-related organelles, and their role in sustaining cellular homeostasis.

## Introduction

Eukaryotic cells contain membrane-confined organelles that enable them to compartmentalize specialized biochemical reactions like ATP production, lipid breakdown or protein degradation, performing them at specific cellular locations. To fully benefit from the clear advantages of such specialization, organelles within a cell must communicate, and exchange information and metabolites. Indeed, it is now well accepted that such interactions are necessary to maintain cell homeostasis. Communication between organelles does not necessarily require physical contacts, as information between organelles can be transmitted through transfer of specific metabolites. However, membrane contact sites (MCSs) represent a fast and efficient way to communicate. Such interorganelle physical contacts are currently the focus of much study and they have been associated to many cellular processes, such as signaling, the regulation of calcium homeostasis, lipid metabolism, and organelle localization and dynamics (Helle et al., 2013; Daniele and Schiaffino, 2014). Interactions between organelles are also very important for protein quality control, since misfolded, oxidized and obsolete proteins are dangerous to the cell, and they must be handled suitably to maintain correct proteostasis. This problem is resolved in the cell by organelles working together in a complex and coordinated network (Baixauli et al., 2014; Gottschling and Nyström, 2017).

Mitochondria and lysosomes are at the center of devastating genetic diseases. Research into mitochondrial and lysosome-originated diseases has focused on trying to understand the molecular pathology of the affected organelle. However, it was recently discovered that genetic defects in one type of organelle may lead to an impairment of the other. Specifically, genetic disruption of mitochondria produces a secondary impairment of lysosomes, and genetic defects in lysosomes can lead to mitochondrial dysfunction, highlighting the functional connection between these two organelles.

Mitochondria are fundamental metabolic organelles in cells, but they also participate in iron and calcium homeostasis, as well as in the regulation of apoptosis, and they are increasingly recognized as key signaling platforms (Friedman and Nunnari, 2014; Chandel, 2015). Like the rest of organelles, mitochondria need to interact with other organelles to carry out their functions, exchanging material and transmitting signals responsible for regulating metabolism, intracellular signaling and cell maintenance (Murley and Nunnari, 2016). This communication can be established in different ways, such as vesicular transport (as initially revealed for organelles within the secretory pathway), the exchange of metabolites or signaling molecules by diffusion, or via direct physical contacts.

The best characterized MSCs are the physical contacts established between the mitochondria and endoplasmic reticulum (ER). These contacts are named mitochondrial-associated membranes (MAMs) and they are critical for many intracellular processes, particularly the regulation of calcium homeostasis, lipid metabolism, mitochondrial fission or regulation of mitochondrial DNA synthesis and division (Friedman et al., 2011; Bravo-Sagua et al., 2014; Lewis et al., 2016). These contacts allow the flux of calcium between the two organelles, coordinating ATP production and facilitating mitochondrial calcium buffering. Under specific conditions, the distance between the mitochondria and ER may vary, affecting their communication and organelle function. For example, tight junctions between mitochondria and ER can cause mitochondrial calcium overload, and mitochondrial membrane permeabilization, driving the cell into an apoptotic programme (Csordás et al., 2006).

Interestingly, defects in MAMs, or in their homeostasis, have been associated with certain diseases, highlighting the importance of the contacts between organelles for controlling both cell and organism homeostasis. Obesity induces an increase of MAMs in the liver, which compromises mitochondrial activity and drives oxidative stress (Arruda et al., 2014; Ma et al., 2017). In obese animals, experimental induction of MAMs deteriorates metabolic homeostasis, while their disruption improves the oxidative capacity of mitochondria (Arruda et al., 2014). MAMs have also been linked to inflammation and there is evidence suggesting that MAMs are involved in triggering an inflammatory response to oxidative stress. Inducers of reactive oxygen species (ROS) activate the inflammasome, driving its localization to MAMs (Zhou et al., 2011). MAMs have been related to neurodegenerative diseases (Manfredi and Kawamata, 2016; Paillusson et al., 2016), such that their frequency and activity are enhanced in both familial and sporadic Alzheimer's disease patients (Area-Gomez et al., 2012). Reduced numbers of ER–mitochondria associations and alteration in Ca^2+^ exchange between these two organelles have been observed in two different amyotrophic lateral sclerosis (ALS) mouse models (Paillusson et al., 2016). The loss of MAM homeostasis has also been linked to cancer, diabetes and pulmonary arterial hypertension (Sutendra et al., 2011; Giorgi et al., 2015; Ma et al., 2017; Rodríguez-Arribas et al., 2017).

The endolysosomal system is a highly dynamic membrane compartment that is critical in the maintenance of cellular homeostasis. The endolysosomal system is made up of several membrane-bound organelles, such as early endosomes, multivesicular bodies (MVBs), lysosomes, lysosome-related organelles (LROs) and other specialized organelles (Klumperman and Raposo, 2014). During endosome maturation, inward budding of the limiting membranes forms small intraluminal vesicles (ILVs) in organelles referred to as MVBs. Endosomes, MVBs and lysosomes regulate signaling, secretion, and the degradation of receptors and other cellular components. MVBs can fuse with the plasma membrane and release their ILVs into the extracellular environment as exosomes, or alternatively, they can fuse with lysosomes for their content to be degraded. Besides degradation, lysosomes carry out many other critical functions, acting as acidic calcium stores that release calcium in response to different stimuli, secreting their content through exocytosis, or repairing the plasma membrane (Perera and Zoncu, 2016). Furthermore, lysosomes can sense the metabolic state of the cell, as it occurs in conditions of starvation or stress, in which mTORC1 is activated and released from the lysosomal membrane, triggering a transcriptional response to increase lysosome biogenesis and activity (Roczniak-Ferguson et al., 2012; Settembre et al., 2013; Ballabio, 2016).

This review will explore some of the more recent findings regarding the communication between mitochondria and the endolysosomal system. Physical contacts between lysosomes and mitochondria are a recent discovery due to the advances in microscopy techniques and molecular biology, and they have mainly been studied in yeast. Beside these physical contacts, mitochondrial derived vesicles (MDVs) represent another means of achieving molecular transfer between mitochondria and the endolysosomal system. Although different modes of communication between these organelles have been identified, their biological meaning is often not yet clear.

## Mitochondria and vacuole interactions in yeast

Yeast has been a powerful model to study the connections between organelles. The vacuole is the equivalent of the lysosome in yeast, and the first description of a functional connection between the vacuole and mitochondria supported a role in lipid metabolism. Indeed, altered cardiolipin synthesis caused vacuolar damage (Chen et al., 2008). Cardiolipin is an anionic phospholipid predominantly found in the mitochondrial inner membrane and its dissociation from mitochondria leads to mitochondrial dysfunction. Deleting cardiolipin synthase, the enzyme responsible for cardiolipin synthesis in mitochondria, causes several defects in vacuoles and it affects cell survival (Chen et al., 2008). Yeast carrying a mutation in cardiolipin synthase have defective vacuolar morphology, decreased v-ATPase activity and vacuolar basification. Hence, cardiolipin plays a key role in the functional crosstalk between mitochondria and vacuoles. Although the mechanism underlying this crosstalk is not clear, it seems to involve a perturbation of ion homeostasis since deletion of the Na^+^/H^+^ exchanger ameliorated the vacuolar defects.

**Figure 1:**
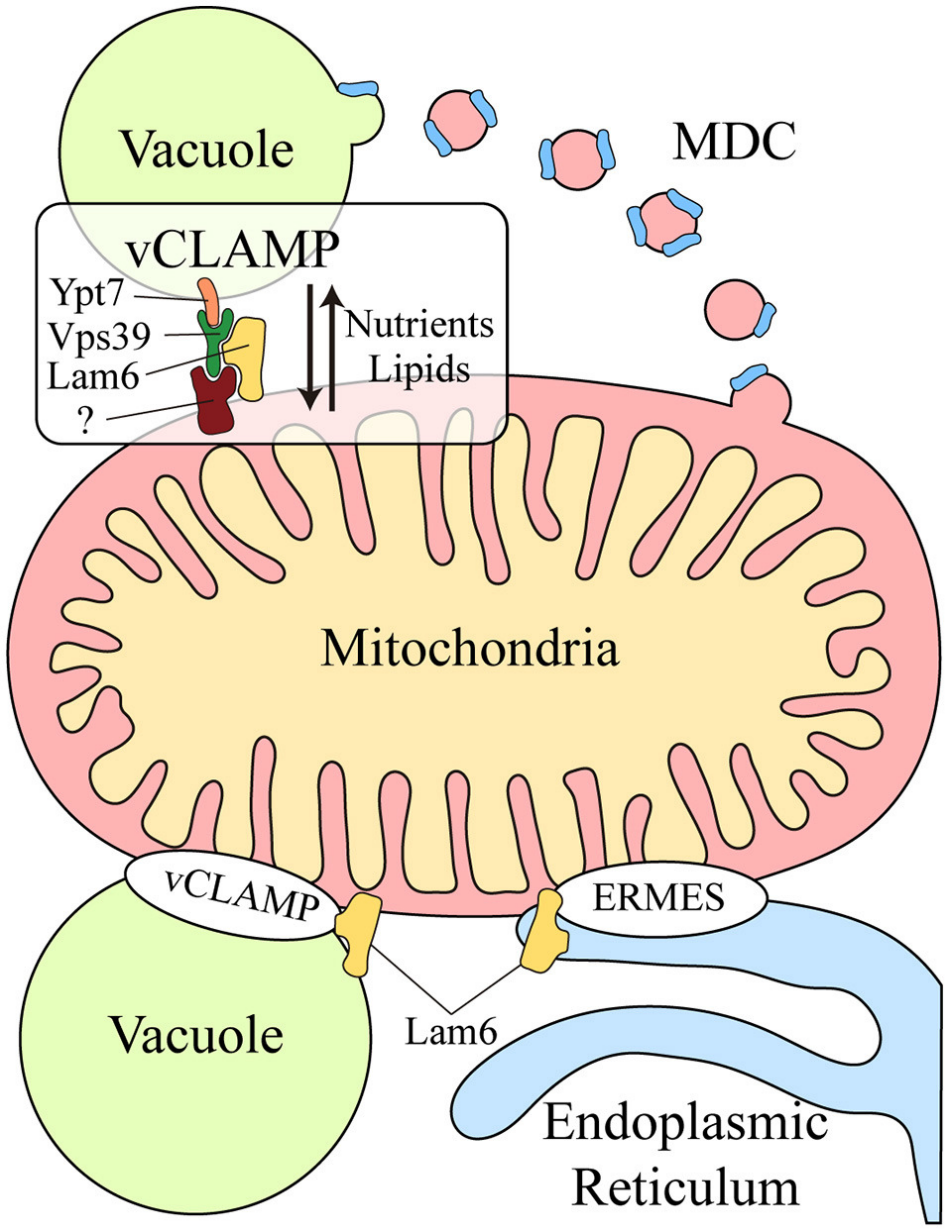
Crosstalk between the mitochondria and lysosomal vacuole in yeast. Two different mechanisms exist for molecular exchange between the mitochondria and lysosomal vacuole: vesicular transport and physical contacts. Mitochondrial membrane proteins can be transported from the mitochondria to the vacuole through the formation of the mitochondrial derived compartment (MDC), particularly for their degradation by autophagy. This mechanism is a protective pathway to preserve mitochondrial integrity in times of stress. In addition, mitochondria and the lysosomal vacuole establish physical contacts, the vacuole and mitochondria patch (vCLAMP), which involves the Vps39, Ypt7, and Lam6 proteins and an unidentified mitochondrial component. This connection participates in the exchange of nutrients and lipids between these organelles. Lam6 is also present in endoplasmic reticulum-mitochondria encounter structure (ERMES), the physical contact between mitochondria and the endoplasmic reticulum. The presence of Lam6 in both mitochondrial contacts makes their co-regulation possible. Hence, vCLAMP is more extensively distributed in an ERMES mutant and conversely, there are more ERMES when vCLAMP is impaired. MDC, Mitochondrial derived compartment; vCLAMP, vacuole and mitochondria patch; ERMES, endoplasmic reticulum-mitochondria encounter structure; (?), unidentified mitochondrial component of vCLAMP.

Another functional correlation between the vacuole and mitochondria was discovered during yeast aging. Aged yeast exhibit mitochondrial fragmentation, loss of mitochondrial DNA in their progeny and increased levels of mitochondrial ROS (Lam et al., 2011; McFaline-Figueroa et al., 2011). Two genes have been identified that are related to aging: *VMA1*, encoding a subunit of the v-ATPase, and *VPH2*, an integral membrane protein of the ER required for v-ATPase assembly. Overexpression of *VMA1* and *VPH2* decreases the vacuolar pH, delaying the mitochondrial dysfunction associated with aging. This pro-longevity effect is related to the capacity of the vacuole to store neutral amino acids rather than to its degradative function (Hughes and Gottschling, 2012). Reduced vacuolar acidity in aged yeast also remodels the mitochondrial proteome (Hughes and Gottschling, 2012). Upon loss of vacuolar acidity, specific membrane proteins from mitochondrial membranes are sorted into a mitochondrial-derived compartment (MDC) (Figure 1). This membrane compartment is then released by fission and transported to the vacuole for protein degradation by autophagy. Proteins are selectively incorporated into the MDC and this requires the mitochondrial import receptors Tom70 and Tom71. This mechanism represents a protective pathway to preserve mitochondrial integrity in times of stress (Hughes et al., 2016).

Two parallel studies demonstrated that physical contacts exist between mitochondria and the yeast vacuole, known as the vacuole and mitochondria patch (vCLAMP) (Figure 1). By screening yeast mutants, two genes were found that affect the formation of the physical contact between the ER and mitochondria, the endoplasmic reticulum-mitochondria encounter structure (ERMES) (Elbaz-Alon et al., 2014). Deletion of the two proteins encoded by these genes, Vps39 and Vam7, that are involved in fusion at the vacuole (Ungermann, 2015), causes an increase in the number of ERMES, although they do not act directly on these structures. Vps39 was previously identified as part of the homotypic fusion and protein sorting (HOPS) tethering complex and it was localized to contact sites between the vacuole and mitochondria (Elbaz-Alon et al., 2014). In a parallel study, physical contacts between mitochondria and vacuoles were discovered by electron microscopy and while Vps39 was implicated in this interaction, Ypt7 was identified in the vacuolar part of the contact (Hönscher et al., 2014) (Figure 1). In both cases, a relationship between ERMES and vCLAMP was seen to exist, as confirmed by an increase in the number of ERMES when the vCLAMP is impaired. Conversely, there are more vCLAMPs when ERMES are disrupted. Notably, yeast mutants for both mitochondrial contacts are lethal, demonstrating that both vacuolar and ER contacts with mitochondria contribute to mitochondrial function, possibly by carrying out partially redundant functions (Elbaz-Alon et al., 2014; Hönscher et al., 2014). These connections are necessary to mediate the transport of phospholipids between the mitochondria and endomembrane system during phospholipid synthesis, a process that requires both organelles. Moreover, vCLAMPs are also enriched for specific ion and amino acid transporters, such that the activity of vCLAMPs may extend beyond lipid transport to nutrient sensing and utilization (Elbaz-Alon et al., 2014). Interestingly, the formation of these contacts depends on the metabolism of the cell. Yeast grown on glucose have more contact sites between mitochondria and vacuoles, and this number is reduced when they are grown in glycerol (Hönscher et al., 2014). Hence, ERMES and vCLAMPs are regulated in response to the metabolic status of cells, suggesting that both contacts are implicated in similar activities but under different conditions, i.e., respiratory vs. fermentative metabolism.

The coordination of these different contact sites is further revealed by studying Lam6, a novel protein that regulates both contact sites in yeast (Elbaz-Alon et al., 2015). Lam6 is present at both ERMES and vCLAMP contact sites (Figure 1), although it is not essential for the formation of these contacts and its absence does not affect their integrity. Rather, Lam6 can extend the contacts between organelles and indeed, vCLAMP is extended in an ERMES mutant but this enlargement does not occur when Lam6 is deleted. Likewise, there are no more ERMES when vCLAMP is impaired in the absence of Lam6, proof of that Lam6 mediates the crosstalk between different organelle contacts in yeast, and in particular that of ERMES and vCLAMP (Elbaz-Alon et al., 2015).

## Mitochondria-endolysosomal system crosstalk in mammals

**Figure 2:**
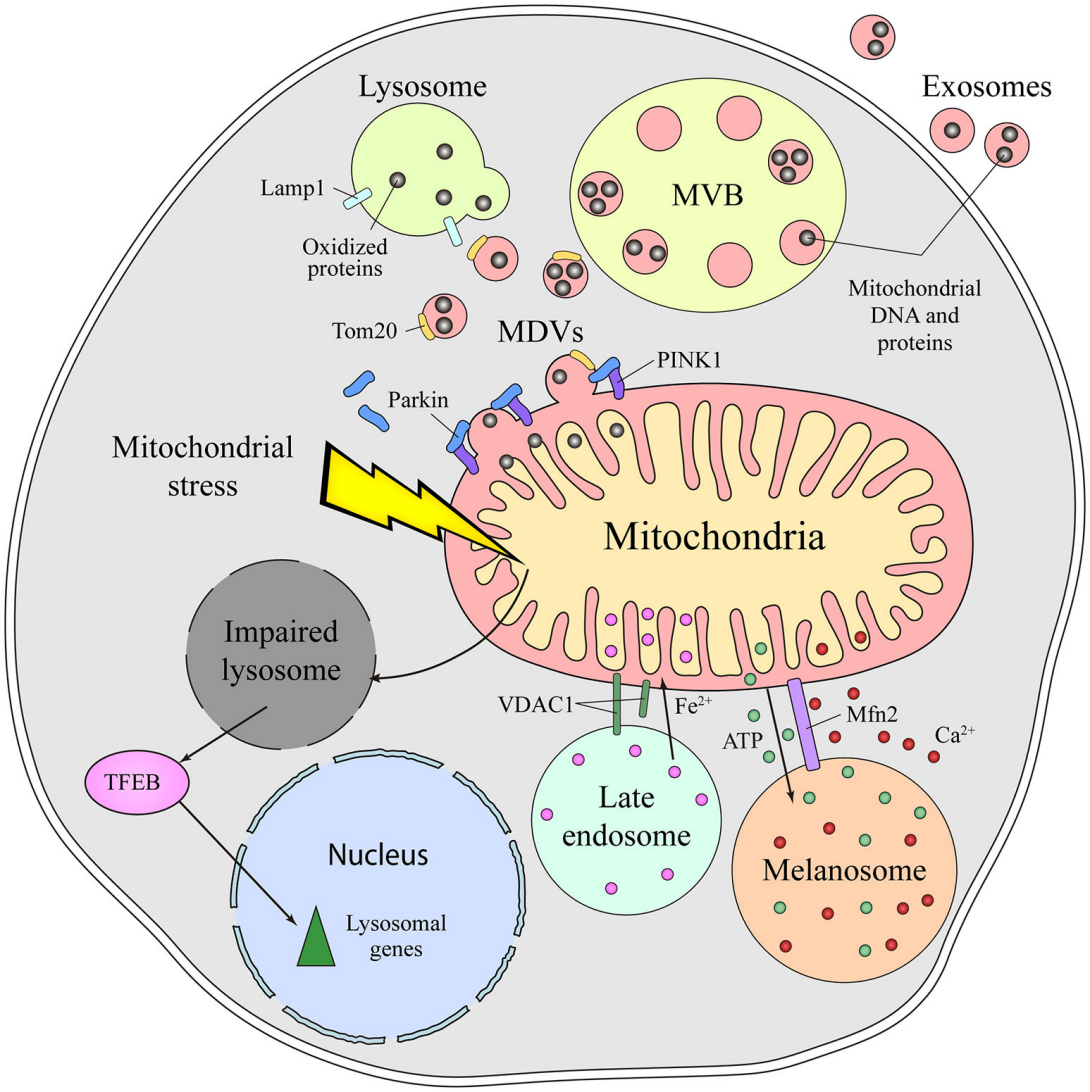
Different means of communication between the mitochondria and lysosome in mammals. Emerging evidence supports the existence of intense crosstalk between the mitochondria and the endolysosomal compartment in mammals. Functional stress or dysfunction of one organelle affects the other. Thus, mitochondrial stress induces a secondary lysosomal dysfunction, which produces activation of TFEB and a transcriptional response associated with lysosomal biogenesis. Additionally, under conditions of stress, mitochondrial derived vesicles (MDVs) are formed in a process dependent on parkin and PINK1. These MDVs traffic obsolete mitochondrial proteins into the endolysosomal system for their degradation, a fast response established to remove oxidized proteins. Once in the endolysosomal route, the mitochondrial content can be degraded by lysosomal enzymes or released to the extracellular milieu via exosomes. Physical connections between the mitochondria and lysosome or melanosome are required for local ATP supply, Ca^2+^ homeostasis, Fe^2+^ transport and to process VDAC1. Mfn2 regulates the mitochondria-melanosome physical connection. MVB, Multivesicular body; MDVs, Mitochondrial-derived vesicles; PINK1, PTEN-induced kinase 1; VDAC1, mitochondrial voltage-dependent anion channel isoform 1; Mfn2, Mitofusin2; TFEB, Transcription Factor EB.

The molecular mechanisms mediating the communication between mitochondria and lysosomes in mammalian cells remain unclear. An initial mode of communication was discovered when mitochondria were shown to form specific vesicles targeted to the endolysomal system (Soubannier et al., 2012). The protein cargo of MDVs is selectively incorporated, and it may be limited to elements at the outer membrane or it might also include inner membrane and matrix components (Figure 2). The molecular mechanism that drives MDVs formation is different from mitochondrial fission, since MDVs are formed in Drp1-silenced cells, Drp1 being a key protein in mitochondrial fission (Soubannier et al., 2012). Parkin induces the PINK1-dependent formation of MDVs under conditions of mitochondrial stress, which are enriched for mitochondrial specific proteins like Tom20 that are targeted to endolysosomal system for degradation (McLelland et al., 2014). These MDVs are originally targeted to the MVB and they finally fuse with lysosome. Such vesicle formation may be a rapid response to mitochondrial stress, shuttling oxidized cargo to lysosomes in order to preserve the integrity of the organelle (McLelland et al., 2014; Sugiura et al., 2014). MDVs have also been reported to be involved in mitochondrial quality control upon acute stress in the cardiac system (Cadete et al., 2016).

Autophagy is the process of removing cellular components through their encapsulation in a double-membrane structure followed by degradation in lysosomes. In particular, the selective removal of damaged mitochondria by autophagy is known as mitophagy. Mitophagy, like MDVs formation, is parkin and PINK1 dependent (Youle and Narendra, 2011; Roberts et al., 2016). However, MDVs are also formed when essential components of autophagy are silenced like Atg5, Beclin-1 or Rab9. Moreover, MDVs do not co-localize with LC3, an autophagosome marker, demonstrating that they are independent of a canonical autophagy process (Soubannier et al., 2012; McLelland et al., 2014). Both MDVs and mitophagy are quality control mechanisms to handle damaged mitochondria. Nevertheless, given that MDVs formation is a faster and less drastic process and it is independent of the classical mitophagy machinery, it could be the first response to mitochondrial stress preceding mitophagy (McLelland et al., 2014; Sugiura et al., 2014).

Mitochondrial dysfunction provokes lysosome impairment, representing a functional connection between the mitochondria and lysosome (Figure 2). Deletion of mitochondrial transcription factor A (TFAM) has been used as a model of mitochondrial dysfunction (Vernochet et al., 2012; Viader et al., 2013), as TFAM is essential for the replication and transcription of mitochondrial DNA (mtDNA) (Larsson et al., 1998). In the absence of TFAM, there is less mtDNA, fewer transcripts encoding mtDNA genes and an impairment of the electron transport chain (Gustafsson et al., 2016). Upon deletion of TFAM in T cells, the impairment of mitochondria augments the number of lysosomes. However, lysosomal activity is profoundly impaired, provoking an accumulation of sphingomyelin and autophagy intermediates, and triggering inflammatory responses (Baixauli et al., 2015). The increased lysosomal mass was caused by the activation of the transcription factor TFEB (Transcription Factor EB), a master transcription factor controlling lysosome biogenesis. Increasing the cellular levels of NAD^+^ by addition of a NAD^+^ precursor improved lysosomal function in this model, evidence that the reduced NAD^+^ levels in cells with impaired mitochondrial function causes lysosome dysfunction. Indeed, disturbing mitochondrial function in neurons by genetic deletion of AIF, OPA1 or PINK1, or with specific mitochondrial inhibitors, provokes morphological alterations to lysosomes (Demers-Lamarche et al., 2016). Upon such mitochondrial alterations, lysosomes become larger and they accumulate lysosomal vacuoles, although neurons appear healthy and viable. A higher lysosomal pH, dampened activity of lysosomal enzymes and an accumulation of substrates of autophagy were also registered in these conditions. Antioxidant treatment partially rescued these lysosomal defects, suggesting that excess of mitochondrial ROS triggers lysosome dysfunction (Demers-Lamarche et al., 2016).

More recently, another functional connection between mitochondria and lysosome was discovered. Pharmacologically impaired mitochondria affect lysosome biogenesis, enhancing the expression of certain lysosomal proteins: Lamp1, a lysosomal membrane protein; and lysosomal enzymes for degradation, such as acid alpha-glucosidase and cathepsins. This effect on lysosome activity is dependent on the duration of the mitochondrial damage. Accordingly, some lysosomal genes are upregulated after 1 week of treatment but their expression is downregulated after 4 weeks of treatment, a pattern that is also observed in fibroblasts obtained from patients with complex I deficiency and in mouse embryonic fibroblasts from a mouse model of mitochondrial malfunction (Fernández-Mosquera et al., 2017). The induction of lysosomal biogenesis in early mitochondrial damage is TFEB-dependent and it requires AMPK signaling, representing a response to mitochondrial stress, increased autophagy flux and the capacity to remove damaged mitochondria (Fernández-Mosquera et al., 2017).

Besides this functional and vesicular connection, direct interorganelle contacts between mitochondria and the endolysosomal compartment have been identified in mammals, specifically in hypoxic cells and erythrocytes (Sheftel et al., 2007; Hamdi et al., 2016). In conditions of hypoxia, mitochondria are hyperfused and they establish physical contacts with late endolysosomes (Figure 2). Such interorganelle contact may be important for the cleavage of VDAC1 by a cysteine protease mainly located in endolysosomes. Cleaved VDCA1 has a different conformation, which protects the mitochondria from autophagy and increases metabolic efficiency (Brahimi-Horn et al., 2015). In erythrocytes, interactions between the mitochondria and late endosomes facilitate the transport of iron between these two organelles (Sheftel et al., 2007; Hamdi et al., 2016).

## Physical connections between mitochondria and melanosomes

Lysosome related organelles (LROs) represent a heterogeneous set of organelles that possess some features common to lysosomes but that are cell type-specific. Consistent with their distinct functions, LROs vary in composition and morphology, and they can be distinguished by the primary source of their membrane and their intralumenal content. Some of the LRO's contents are derived from the endolysosomal system, and most of them contain lysosomal proteins and have a low lumenal pH (Raposo et al., 2007).

Melanosomes are LROs of pigmented cells devoted to the synthesis, storage and transport of melanins. Melanosomes are considered biological models of organelle biogenesis and motility. They originate from endosomal precursors and subsequently, they mature through the progressive deposition of melanin and they are transported toward the cell periphery (Raposo et al., 2001). Contacts between mitochondria and melanosomes are visible by electron microscopy and tomography, and these connections affect ca. 1% of melanosomes (Daniele et al., 2014). The formation of these contacts is not based on membrane fusion but on the establishment of fibrillar bridges between the two organelles (Figure 2). Mitofusin 2 is thought to play an essential role in these contacts, a surprising new function of this protein that was originally identified as part of MAMs (de Brito and Scorrano, 2008). Mitochondria-melanosome contacts are more abundant in the perinuclear area where new melanosomes are generated and, indeed, stimulating melanosome biogenesis was found to enhance the presence of these contacts. Moreover, these contacts could mediate the ATP synthesis necessary for the maturation and acidification of melanosomes, as well as influencing other events in melanosomes, like melanin synthesis and the exchange of small molecules. Melanosomes are considered acidic calcium stores, indicating that they may also be important in calcium signaling. Due to the antioxidant and free radical scavenging roles of melanin, this connection might be involved in controlling the redox-status of melanocytes.

## Mitochondrial molecules are secreted to the extracellular media by exosomes

Exosomes are extracellular vesicles that are secreted by all cell types and that are derived from MVBs upon their fusion with the plasma membrane. The presence of mitochondrial molecules in exosomes represents further indirect evidence of the crosstalk between mitochondria and the endolysosomal system (Torralba et al., 2016). Before being exported to the extracellular milieu, toxic, obsolete or damaged mitochondrial material is loaded into endolysosomes for degradation or extracellular export. Exosomes contain genetic material, mostly non-coding RNA, but also mtDNA (Guescini et al., 2010a,b) (Figure 2). Moreover, mesenchymal stem cells and astrocytes can produce even larger vesicles that contain entire mitochondrial particles and mtDNA (Phinney et al., 2015; Hayakawa et al., 2016). The physiological function behind the transport of mitochondrial DNA and proteins out of the cell remains unclear, as do the molecular events responsible for their loading into vesicles. MDVs or direct interorganelle contacts could represent a rapid mechanism to relieve mitochondrial stress in order to maintain the cell fit, perhaps being employed when other degradation pathways are compromised (Desdín-Micó and Mittelbrunn, 2017).

## Concluding remarks

The study of all facets of interorganelle connections is possible due to new super-resolution imaging and molecular biology techniques, approaches that have shed more light on this emerging and promising field of research. The crosstalk and dependency between organelles could be fundamental to better relate currently misunderstood cell processes. Indeed, secondary effects on related organelles are important in disease pathophysiology and they may represent a starting point to identify novel therapeutic targets to treat human disease. This is especially critical in the case of mitochondrial and lysosomal diseases, devastating pathologies that mostly represent unmet medical needs. Mitochondria and lysosomes (or LROs) interact at different levels and in distinct ways to control cell homeostasis, and these connections are essential for the correct functioning of both organelles. In mammals, communication between organelles has mainly been described at a functional level, observed as secondary damage to lysosomes or LROs when mitochondria are altered, particularly when cells are under stress. Due to the role of lysosomes in degradation, this communication could be a rapid way to firstly reduce mitochondrial stress and preserve mitochondrial integrity. Although physical contact between mitochondria and the endolysosomal compartment has been mainly studied in yeast, recent evidence suggests that this is preserved in mammals. The connection between these organelles could coordinate processes that require the participation of both organelles, such as metabolic adaptation, phospholipid metabolism, regulation of calcium signaling and the control of cellular homeostasis.

## Author contributions

All authors listed have made a substantial, direct and intellectual contribution to the work, and approved it for publication.

### Conflict of interest statement

The authors declare that the research was conducted in the absence of any commercial or financial relationships that could be construed as a potential conflict of interest.
